# Changes in Serum Metabolome Following Low-Energy Diet-Induced Weight Loss in Women with Overweight and Prediabetes: A PREVIEW-New Zealand Sub-Study

**DOI:** 10.3390/metabo14080401

**Published:** 2024-07-24

**Authors:** Bárbara Relva, Linda M. Samuelsson, Iola F. Duarte, Ulrike Fasol, Patrick J. B. Edwards, Mikael Fogelholm, Anne Raben, Sally D. Poppitt, Marta P. Silvestre

**Affiliations:** 1 NOVA Medical School, Faculdade de Ciências Médicas, NMS|FCM, Universidade Nova de Lisboa, 1169-056 Lisboa, Portugal; barbara.c.relva@edu.nms.unl.pt; 2 Smart Foods & Bioproducts Group, AgResearch Ltd., Palmerston North 4442, New Zealand; linda.samuelsson@agresearch.co.nz; 3 Department of Chemistry, CICECO-Aveiro Institute of Materials, University of Aveiro, 3810-193 Aveiro, Portugal; ioladuarte@ua.pt; 4 Faculty of Medical and Life Sciences, Furtwangen University, 78054 Villingen-Schwenningen, Germany; ulrike.fasol@hs-furtwangen.de; 5 School of Natural Sciences, Massey University, Palmerston North 4442, New Zealand; p.j.edwards@massey.ac.nz; 6 Department of Food and Nutrition, University of Helsinki, Unioninkatu 44, 00014 Helsinki, Finland; mikael.fogelholm@helsinki.fi; 7 Department of Nutrition, Exercise and Sports, University of Copenhagen, Nørre Allé 51, DK-2200 Copenhagen, Denmark; ara@nexs.ku.dk; 8 Department for Clinical and Translational Research, Copenhagen University Hospital, Steno Diabetes Center Copenhagen, Blegdamsvej 9, DK-2100 Herlev, Denmark; 9 Human Nutrition Unit, School of Biological Sciences, Department of Medicine, University of Auckland, 18 Carrick Place, Auckland 1024, New Zealand; 10 CINTESIS@RISE, NOVA Medical School, Faculdade de Ciências Médicas, NMS|FCM, Universidade Nova de Lisboa, 1169-056 Lisboa, Portugal

**Keywords:** low-energy diet, metabolomics, NMR spectroscopy, obesity, polar metabolites, prediabetes, weight loss

## Abstract

As obesity develops, metabolic changes increase the risk of non-communicable diseases such as type 2 diabetes (T2D). Weight loss is crucial for improving health in T2D and cardiometabolic conditions. However, weight loss rates vary between individuals, even with identical diets or energy restrictions, highlighting the need to identify markers or predictors of weight loss success to enhance intervention outcomes. Using nuclear magnetic resonance (NMR) spectroscopy-based metabolomics, we investigated the change in serum polar metabolites in 28 women with overweight or obesity and prediabetes who completed an 8-week low-energy diet (LED) as part of the PREVIEW (PREVention of diabetes through lifestyle intervention and population studies in Europe and around the World) clinical trial. We aimed to characterize the metabolic shift in substrate oxidation under fixed energy intake (~4 MJ/day) and its relation to weight loss success. Nine of the thirty-four serum metabolites identified significantly changed during the LED phase: 3-hydroxybutyrate, *O*-acetylcarnitine, 2-hydroxybutyrate, mannose, dimethyl sulfone and isobutyrate increased, whilst choline, creatine and tyrosine decreased. These results confirmed a shift towards lipid oxidation, but no metabolites predicted the response to the LED-induced weight loss. Further studies in larger populations are required to validate these metabolites as biomarkers of diet exposure.

## 1. Introduction

Obesity is a growing public health problem [1]. Recent updates from the World Obesity Atlas show that worldwide, in 2020, more than 2.6 billion people aged over 5 years were overweight, and 988 million were obese [2]. Given the rise in risk of type 2 diabetes (T2D) and cardiovascular disease caused by obesity [3,4] and the difficulty experienced by individuals overweight in effectively maintaining weight loss [5], there is a need to understand the molecular determinants of weight change. Several different factors contribute to body weight homeostasis in humans [6], including biological factors, such as genetic and epigenetic, physiological, behavioural, sociocultural and environmental factors [5,7]. Moreover, as obesity develops, a number of metabolic changes occur, which may not completely reverse when weight is lost [8].

Metabolomics, defined as a technology aimed at measuring/profiling changes in the concentrations of metabolites present inside a cell, tissue, or organism in response to a genetic variation, pathophysiological stimuli, or environmental factors, has been used for metabolic profiling and biomarker discovery for a number of clinical conditions [4]. By identifying early biomarkers of disease, metabolomics can provide a better understanding of disease progression and metabolic pathways [4]. Also, metabolomics can provide background knowledge of the underlying mechanisms of obesity and associated diseases such as T2D [9] and thereby be helpful for correlating metabolite changes with the effect of external influencing factors such as diet, drugs, or contaminants [10].

Recently, a number of studies have focused on the relationship between metabolite profile and weight loss [11,12,13,14,15,16], but evidence for the changes that occur during metabolic adaptations to weight loss is still scarce [17]. For instance, elevated concentrations of branched-chain amino acids (BCAAs) (leucine, isoleucine and valine) and aromatic amino acids (phenylalanine, tyrosine, tryptophan and methionine), as well as some of their tissue metabolites, have been detected in individuals with obesity and those with T2D, whereas glutamine and glycine concentrations were decreased [14,17]. Several authors have shown that weight loss is associated with changes in lipid species [11,12,13,18,19], such as ketone bodies (acetoacetate and 3-hydroxybutyrate) [16], lysophosphatidylcholines (LPCs), phosphatidylcholines (PCs) and sphingomyelins (SM) [13] in adults with overweight or obesity following a low-energy diet (LED), with consequent decrease in body weight and adiposity. In the DiOGenes intervention, Stroeve et al. reported that 57% of the variance of weight loss success was predicted by baseline metabolic parameters [16]. Energy-related metabolites, such as acetoacetate, triacylglycerols, PCs, specific amino acids, creatine and creatinine, were the best predictors of weight loss in individuals with morbid obesity [16]. Geidenstam et al. found that lower baseline concentrations of xylitol were predictive of a greater decrease in body mass index (BMI) and ≥10% weight loss [14].

Intermediary hyperglycaemia, also known as prediabetes, is a term commonly used to describe individuals with impaired fasting glucose (IFG) and/or impaired glucose tolerance (IGT) and indicates a higher risk of developing T2D and diabetes-related complications [20]. There are a series of metabolomic studies carried out in cohorts with obesity and T2D which have shown potential predictive biomarkers of disease progression [4,8]. However, identification of biomarkers of early T2D onset prior to clinical diagnosis is crucial in order to define early metabolic derangements associated with impaired glycemic control and ultimately improve prediction, early diagnosis and intervention of the disease at earlier stages [21].

Notably, long term weight loss success is not identical between individuals, with variable response of body weight and adiposity to energy restriction [16,22]. Explained in part by factors such as compliance to diet, this variability is likely also the result of compensatory changes in both physiological and behavioural factors as yet unresolved [23]. Identifying metabolites that can serve as predictive markers of weight loss response may contribute to improving the success of these interventions.

Our current study aimed to investigate metabolic changes that occur in women with overweight or obesity and prediabetes during a period of LED-induced rapid weight loss. Since our research team has previously shown that women and men respond differently to an LED intervention [24], in order to minimise variability between individuals, this was conducted as a single-gender study. We were particularly interested in characterising the metabolic shift in substrate oxidation and how that is related to the efficiency of body weight loss (defined as greater loss per unit energy restriction) [23].

Our data were obtained from PREVention of diabetes through lifestyle intervention and population studies in Europe and around the World (PREVIEW), a multi-centre, 3-year lifestyle intervention in adults with overweight and prediabetes, conducted in eight countries, which aimed to decrease the incidence of T2D [25]. The 3-year randomised controlled trial (RCT) included an initial 8-week LED weight loss phase, with mean body weight loss of ~11% of baseline [24]. The PREVIEW study has previously shown that poor responders to the LED displayed behavioural vulnerabilities, including less favourable responses to hunger and appetite sensations [26]. Here, we investigated the change in polar serum metabolites in response to the LED (first 8 weeks of the trial) in a subset of PREVIEW participants resident in New Zealand, using a non-targeted metabolomics approach.

## 2. Materials and Methods

### 2.1. Participants

Between June 2013 and February 2015, a total of 317 adults with overweight or obesity (BMI > 25 kg/m^2^) and prediabetes, according to the American Diabetes Association (ADA) Criteria [27], were enrolled in the New Zealand arm of the PREVIEW study. Participants were recruited in the Auckland region through adverts in local newspapers, general practitioner (GP) clinics and through the media. All participants gave informed consent for inclusion before they were screened. The study was conducted in full compliance with the relevant requirements of the latest version of the Declaration of Helsinki (59th WMA General Assembly, Seoul, Korea, October 2008) and the ICH-GCP, The International Conference on Harmonisation (ICH) for Good Clinical Practice to the extent that this is possible and relevant. The study protocol was approved by the Health and Disability Ethics Committee (HDEC, 13/NTB/41), Auckland, New Zealand. All information obtained during the trial was handled according to local regulations and the European Directive 95/46/CE (directive on the protection of individuals with regard to the processing of personal data and on the free movement of such data). The trial was registered at clinicaltrials.gov as NCT01777893.

For the purpose of the analysis presented here, a subset of 28 female participants with overweight or obesity and prediabetes, aged between 39 and 60 years, were selected for the metabolomic profiling. Participants were selected based on their ethnicity (Caucasian), age group and sex to avoid having a high number of variables that could bias/confound the results. Participants self-reported not being engaged in competitive sports, with stable body weight (±5 kg) for at least 2 months prior to the study, and no current glucose medications or changes in prescribed medications for 3 months prior to sample collection. Exclusion criteria included diagnosed diabetes, other significant diseases including cardiovascular, liver, gastrointestinal or kidney disease, malignancy, bariatric, or any major surgical procedure in the previous 3 months, systolic blood pressure above 160 mmHg and/or diastolic blood pressure above 100 mmHg, pregnancy, or breastfeeding. A detailed protocol for the intervention has been published elsewhere [25]. All study participants undertook the first phase of the PREVIEW RCT, which consisted of an 8-week weight loss phase, achieved using a complete meal replacement LED program [28].

### 2.2. Study Treatments

The standardised LED consisted of ~4 MJ/day, based on commercial meal replacement powders reconstituted in skimmed milk or water from Cambridge Weight Plan^®^. In total, the LED provided an estimated 3.4 MJ/day (810 kcal/day), of which 43.7 total energy % (en%) from protein (~88.5 g/day), 41.2 en% from carbohydrate (~83.4 g/day) and 15.1 en% from fat (~13.6 g/day). The fibre content was 13.3 g/day. Additionally, participants were recommended to consume psyllium fibre (daily) and sufficient water to avoid gastrointestinal side effects. A maximum of 400 g of non-starchy vegetables could be consumed, such as tomatoes, cucumber and lettuce, making the total energy content approximately 4 MJ (1000 kcal). Those who successfully achieved ≥8% weight loss between baseline and 8 weeks were eligible to continue into the weight maintenance phase over a period of 3 years [28].

### 2.3. Clinical Measurements

The detailed protocols for clinical sample collection and outcome measurements have been described elsewhere [25]. Data and samples were obtained from participants at baseline, week 0 prior to the start of LED (clinical investigation day 1, CID1) and after 8 weeks (CID2). Participants were classified according to their weight loss success at the end of the 8-week LED in order to identify weight loss predictors. Weight loss success was expressed as the percentage of weight change from baseline to 8 weeks, calculated as ((body weight at week 8 − body weight at week 0)/body weight at week 0) × 100). Based on obesity guidelines that state that there are clinically significant improvements for weight loss of 5 to 10% of initial body weight [29] and that LEDs have been shown to achieve weight loss of 10 to 15% [30], two groups were created using a cut point of 10%: highly successful weight loss (≥10%) or moderately/unsuccessful weight loss (<10%).

### 2.4. Blood Collection

Fasting venous blood samples were collected for laboratory measurements, including plasma glucose, serum insulin and lipid profile (total cholesterol, high-density lipoprotein (HDL) cholesterol, low-density lipoprotein (LDL) cholesterol and triglycerides, TG). Laboratory measurements were performed on an Architect ci8200 integrated system (Abbott Laboratories, Abbott Park, IL, USA) at the National Institute for Health and Welfare, Helsinki. Homeostatic model assessment for insulin resistance (HOMA-IR) was calculated as a proxy for insulin resistance. The equation used was (fasting insulin (mU/L) × fasting plasma glucose (mmol/L))/22.5. Samples were stored at −80 °C for later batch analyses.

### 2.5. Metabolomics

Non-targeted metabolomic profiling of polar serum metabolites using ^1^H-NMR spectroscopy was carried out at the Massey University bioNMR facility in Palmerston North, New Zealand.

#### 2.5.1. Chemicals

Acetone (pro analysis grade) was purchased from Merck, Darmstadt, Germany. 2,2-Dimethyl-2-silapentane-5-sulfonate or 3-(trimethylsilyl)-1-propanesulfonic acid sodium salt (DSS, purity 97%) was purchased from Sigma-Aldrich, Saint Louis, MO, USA. Deuterated water (D_2_O, 99.8 atom% purity) was purchased from Cambridge Isotope Laboratories Inc., Tewksbury, MA, USA. Phosphate buffered saline (PBS) was prepared fresh on the day in MilliQ water and had a final concentration of 137 mM NaCl, 2.7 mM KCl, 10 mM Na_2_HPO_4_ and 1.8 mM KH_2_PO_4_. The final pH was adjusted to 7.4.

#### 2.5.2. Sample Preparation

Both serum samples (week 0 and week 8) from each of the 28 participants were randomised and thawed on ice. Samples were prepared by a modified method based on an existing protocol [31] to identify polar metabolites. A volume of 1.2 mL ice-cold acetone was added to 400 μL of serum in microcentrifuge tubes and the samples were vortexed for 10 s and then kept on ice for 10 min. Samples were then centrifuged at 10,000× *g* for 10 min at 4 °C, and 1.2 mL of the supernatant was transferred to new tubes. The solvent was evaporated overnight by vacuum centrifugation. Residues were stored at −20 °C. On the day of the NMR analysis, residues were resuspended in 595 μL PBS by vortexing for 10 s. Samples were centrifuged at 10,000× *g* for 10 min at 20 °C to remove any remaining particulate matter, and 585 μL of the supernatant was mixed with 65 μL internal standard solution (containing 5 mM DSS in D_2_O, pH 6.5) and transferred to 5 mm NMR tubes. In addition, two pooled samples, one for week 0 and one for week 8, were prepared by mixing 20 μL of serum from each sample within each treatment group before acetone extraction, as described above. These samples were used for metabolite identification using 2D NMR techniques.

#### 2.5.3. NMR Spectroscopy

Spectra of the polar serum metabolites were acquired on a Bruker Avance 700 MHz NMR spectrometer (Bruker-Biospin, GmbH, Rheinstetten, Germany) operating at 700.13 MHz and equipped with a three-channel inverse detection cryo-probe. All spectra were recorded at 298 K, and temperature calibration was conducted using the separation of the residual ^1^H signals from a standard sample of methanol-*d_4_*. Suppression of the water signal was achieved via pre-saturation at the water offset frequency using a field strength of 100 Hz. One-dimensional (1D) ^1^H spectra were recorded on individual serum samples for metabolomic profiling using the standard Bruker ‘noesygppr1d’ pulse sequence, using the following parameters: spectral width (SW)—8.33 kHz (11.90 ppm) and 58k points; acquisition time—3.50 s; recycle delay—1.50 s; and number of scans—128 plus 4 dummy scans. The following two-dimensional (2D) NMR spectra were acquired for the two pooled serum samples for metabolite identification: one for week 0 and one for week 8. ^1^H-^13^C heteronuclear single quantum coherence (HSQC) spectra were acquired using the ‘hsqcetgpsisp2.2’ pulse sequence with an SW of 8.39 kHz (11.98 ppm) in the F2 domain and 2048 points (acquisition time: 0.122 s) and an SW of 33.4 kHz (190.0 ppm) and 512 points in the F1 domain (acquisition time: 7.65 ms). Data acquisition of 72 scans per row was preceded by 64 dummy scans. ^1^H-^1^H total correlation spectroscopy (TOCSY) spectra were recorded using the standard Bruker ‘mlevgpphw5’ pulse sequence with an SW of 8.39 kHz (11.98 ppm) in the F2 domain and 4096 points (acquisition time: 0.24 s) and an SW of 8.40 kHz (12.00 ppm) in the F1 domain and 400 points (acquisition time: 0.024 s). A mixing time of 60 ms was used. Data acquisition of 48 scans per row was preceded by 64 dummy scans.

#### 2.5.4. Data Processing and Metabolite Quantification

One-dimensional (1D) NMR spectra of the polar serum extracts were processed, and metabolites were quantified using Chenomx NMR Suite 8.0 (Chenomx Inc., Edmonton, AB, Canada). Spectra were phased, baseline correction was performed (using the Whittaker-Smoother algorithm), chemical shape indicator (DSS) and pH calibration were performed. Finally, the region around the water peak was removed. Metabolites were putatively identified using the Chenomx database; spectra of individual metabolites were profiled, and the peak areas of the metabolite peaks were related to the area of the internal standard (DSS) to calculate absolute metabolite concentrations.

Putative metabolite IDs were confirmed using the 2D NMR spectra from the two pooled samples. The ^1^H-^13^C HSQC and ^1^H-^1^H TOCSY spectra of the pooled samples were compared with the corresponding spectra of individual metabolites in the Human Metabolome Database (www.hmdb.ca (accessed on 12 June 2024)).

### 2.6. Statistical Analysis

The SPSS version 27 software (IBM/SPSS, Chicago, IL, USA) and R version 4.3.0 [32] software were used for the statistical analysis. Differences in anthropometric, clinical variables and metabolite concentrations at baseline, pre- and post-LED were assessed by paired Student’s *t*-test. The normality of the data was assessed with the Kolmogorov–Smirnov test. The non-parametric Wilcoxon test was used when appropriate. Differences between groups (<10% or ≥10%) were assessed by the Mann–Whitney test. Data were expressed as mean ± SD unless otherwise stated.

Metabolite concentration data were log-transformed and Pareto-scaled prior to analysis using MetaboAnalyst 5.0 (www.metaboanalyst.ca (accessed on 12 June 2024)), and the analysis was conducted on paired data. Principal component analysis (PCA), which is an unsupervised multivariate data analysis method, was used to obtain an overview of the data and detect potential outliers. Partial Least Squares Discriminant Analysis (PLS-DA), a supervised method, was used to maximise the separation between groups and a cross-validation method was used to validate the prediction. Variable Importance in Projection (VIP) scores show the important features identified by PLS-DA and were used to identify the most important features of each component (VIP > 1).

For the association between changes in anthropometric and clinical measures with changes in significant metabolite concentrations, multivariable linear regression models were used, with robust estimates and standard errors (95%) adjusted for age. BMI (kg/m^2^), fat mass (FM) (%), fasting insulin (mU/L), HDL-cholesterol (mmol/L), TG (mmol/L) and waist circumference (cm) were added to the models as independent variables based on the fact that all changed significantly during the weight loss phase. Total cholesterol was not included in the models because it is dependent on other variables already included (LDL-cholesterol, HDL-cholesterol and TG).

To identify baseline metabolites that could predict the weight changes, simple (Model 1) and multiple (Model 2) robust logistic regression models using categorical weight loss (<10% vs. ≥10%) as the dependent variable were used, adjusted for the variables at baseline body weight, fasting insulin, HDL-cholesterol, TG and waist circumference, with estimates and standard errors (95%). The variables included had the same assumption as before, but FM (%) was removed due to multicollinearity. A significance level of 5% was adopted.

While serum glucose was identified and quantified by NMR, these data were excluded from the statistical analyses: serum for NMR analysis was collected in serum gel tubes containing no glucose preservative, which may impact the accuracy of the measured glucose concentrations by this method. For the purpose of the statistical analyses, fasting plasma glucose concentrations analysed using the Architect ci8200 system were used instead.

## 3. Results

### 3.1. Baseline Characteristics of Study Participants

The anthropometric and clinical variables of study participants at baseline pre- (week 0) and post-LED (week 8) are presented in Table 1 and were compared between the two weight loss groups (<10% vs. ≥10%). The mean (±SD) age at enrolment was 50.5 ± 6.1 years, and the baseline BMI was 37.0 ± 5.5 kg/m^2^. Mean weight loss for all participants was 11.2 ± 2.6 kg (*p* < 0.001) (Table 2), with 64.3% of participants losing ≥10% of baseline body weight (Table 1). Post-LED BMI, fat-free mass (FFM), FM, waist and hip circumference, fasting insulin, HOMA-IR, total cholesterol and LDL-cholesterol all significantly decreased in both weight loss groups. In contrast, unexpectedly, fasting plasma glucose and diastolic blood pressure (BP) did not change in either group post-LED (Table 1). Changes in body weight (*p* < 0.001), BMI (*p* < 0.001), waist circumference (*p* = 0.002), waist–hip ratio (*p* = 0.005) and HDL-cholesterol (*p* = 0.021) differed significantly between the two weight loss groups (Table 2).

### 3.2. LED-Dependent Weight Loss Shifts Metabolism Towards Lipid Oxidation

**Figure 1:**
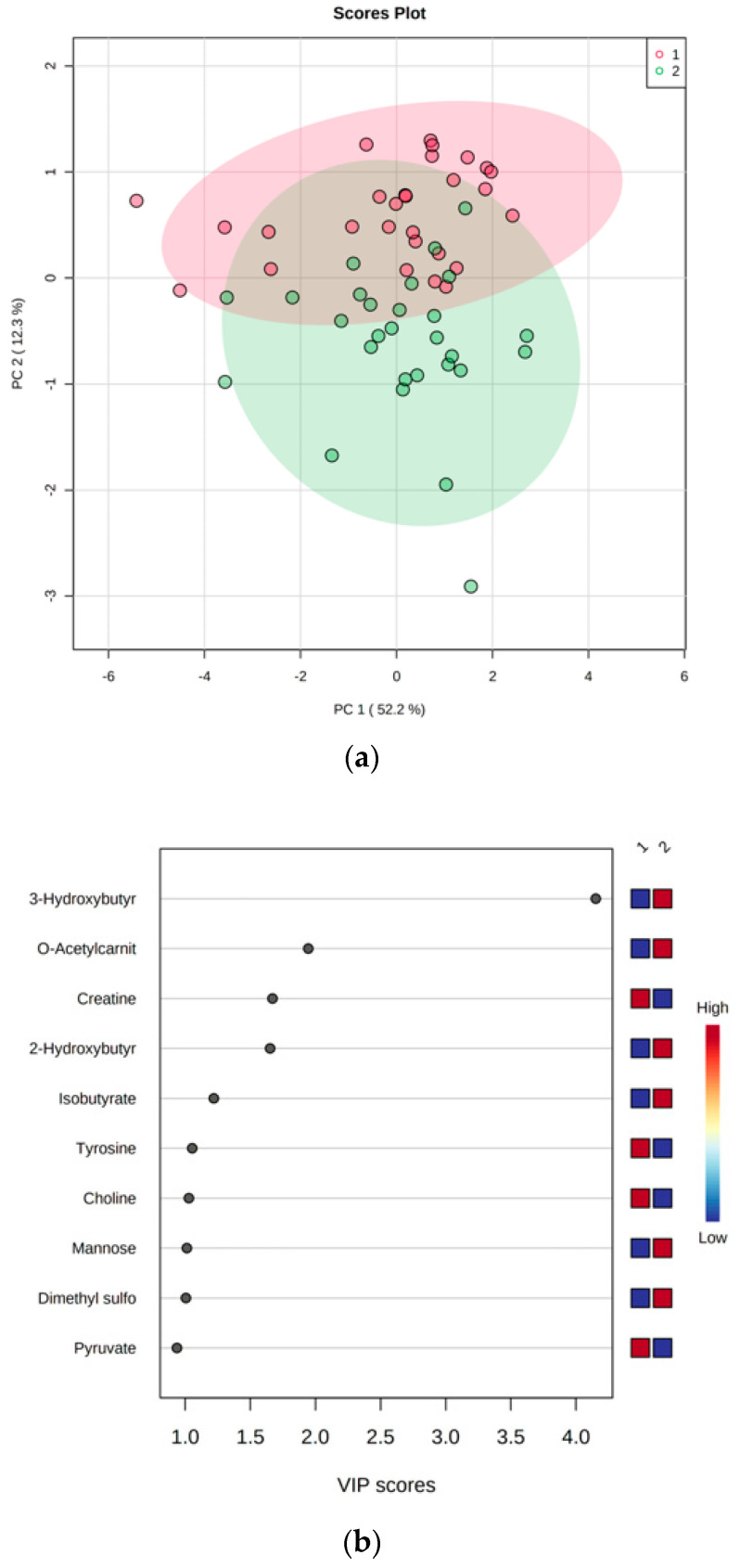
(**a**) The PCA score plot provides an overview of all paired samples and shows some separation between groups. PC 2 explains 12.3% of variance, and PC 1 explains 52.2% of variance. Red symbols—pre-LED (week 0), and green symbols—post-LED (week 8). (**b**) VIP scores of the most important metabolites. Pyruvate has a VIP < 1. The coloured squares represent the relative concentrations of the metabolites at each time point (1—pre-LED week 0; 2—post-LED week 8). Blue represents a low concentration, and red represents a high concentration of the metabolite. Among the significantly altered metabolites, the greatest increase was observed in lipid oxidation-related metabolites (VIP > 1).

From the NMR analysis, 34 polar serum metabolites were identified and quantified pre- (week 0) and post-LED (week 8), with mean serum concentrations reported in Table 3. The PCA score plot gives an overview of the samples and demonstrates a slight difference between groups (Figure 1a). PLS-DA, which uses the classified data to maximise the separation between the two groups, makes this difference more evident (Figure S1, Supplementary Materials). Similar to the results reported in Table 3, VIP scores from the PLS-DA analysis showed nine altered metabolites post-LED (Figure 1b). Among the significantly altered metabolites, the greatest increase was observed in lipid oxidation-related metabolites, including 3-hydroxybutyrate (*p* < 0.001), *O*-acetylcarnitine (*p* < 0.001), 2-hydroxybutyrate (*p* = 0.002) and the short-chain fatty acid (SCFA) isobutyrate (*p* = 0.006). Other metabolites, such as mannose (*p* = 0.003) and dimethyl sulfone (*p* = 0.007), also increased. Choline (*p* = 0.008) and the amino acids creatine (*p* < 0.001) and tyrosine (*p* = 0.005) decreased after the LED-induced weight loss.

### 3.3. Association between Changes in Anthropometric and Clinical Parameters and Changes in Metabolite Concentrations

Associations between changes in clinical variables (BMI; FM (%); fasting insulin; HDL-cholesterol; TG; waist circumference) and changes in the serum metabolite concentrations observed to change significantly from week 0 to week 8 were assessed using multiple linear regression models. The results are presented in Table 4. A decrease of 1% of FM was associated with an increase of 6.1 µM (95% CI: 0.59; 11.62) 2-hydroxybutyrate; 36.7 µM (3.05; 70.28) 3-hydroxybutyrate; 4.1 µM (0.19; 8.06) mannose; and 1.2 µM (0.30; 2.16) *O*-acetylcarnitine. The same negative association occurred such that a decrease of 1 mU/L in fasting insulin was associated with an increase of 23.2 µM (10.85; 35.53) 3-hydroxybutyrate. Likewise, a decrease of 1 mmol/L in TG was associated with an increase of 34.3 µM (2.56; 65.98) 2-hydroxybutyrate; 4.8 µM (0.99; 8.61) choline; and 7.2 µM (1.30; 13.08) *O*-acetylcarnitine.

### 3.4. Baseline Metabolites Do Not Predict Weight Loss

Table 5 shows the logistic regression models of the relationship between metabolite concentrations at baseline and categorical weight loss groups (<10% or ≥10%). No associations were found between baseline metabolite concentrations and weight loss in either model.

## 4. Discussion

Our current study showed that LED-induced weight loss in women with overweight and prediabetes resulted in a shift towards lipid oxidation, as expected, which could be identified through changes in serum polar metabolites. Conversely, weight loss success could not be predicted from baseline metabolites identified prior to the start of the LED. Changes in polar metabolites included increased 2-hydroxybutyrate, 3-hydroxybutyrate, *O*-acetylcarnitine, and decreased choline. Since lipids are one of the main sources of energy for metabolism [4], it is not unexpected that they were the main substrate utilised after weight loss induced by this 8-week LED. Gu et al. reported that hypoenergetic, very low-carbohydrate diets in participants with obesity lead to a greater increase in markers of lipolysis (ketones and free fatty acids, FFAs) together with a greater decrease in markers of lipogenesis and deposition of body fat when compared to hypoenergetic low-fat diets [19]. Thus, the change in serum metabolites in our current study suggests that the increase in lipid oxidation may have been favoured by the low intake (g) of carbohydrates and the consequent reduction in fasting insulin. This was alongside other potentially important changes such as the metabolites dimethyl sulfone and the SCFA isobutyrate, which may, in turn, reflect the high intake of low-fat milk consumed as part of the LED meal replacement regime throughout the 8-week intervention, although this cannot be verified in our current trial. Interestingly, increased isobutyrate has been proposed as a marker of improved insulin sensitivity following weight loss [33], which does align with our finding of decreased HOMA-IR with LED-driven weight loss.

Regarding the metabolites identified in our current trial, in turn, 2-hydroxybutyrate has previously been shown as positively associated with BMI [34] and considered an early marker for dysglycemia and IR [35], independent of sex, age and BMI. This is hypothesised to be due to increased lipid oxidation and oxidative stress [36]. 3-Hydroxybutyrate, a ketone body and final product of FA β-oxidation, also increased in our study and is in line with literature reports that show high concentrations of circulating ketone bodies under energy-restricted and fasted states through increased lipolysis of FA in hepatic mitochondria [37]. In addition, other authors [4,16,19,37] have also reported a significant increase in 3-hydroxybutyrate after weight loss, which may reflect energy homeostasis through increased lipid oxidation.

*O*-Acetylcarnitine is an acetylated form of the AA derivative carnitine. Gu et al. and Newgard et al. reported some key metabolites of FA synthesis and oxidation, including carnitine, as significantly higher in individuals with obesity compared to normal-weight individuals [19,38]. FAs provide energy through β-oxidation, with some evidence that obesity and T2D are associated with a decreased ability to oxidise FA [19]. Increased availability of (non-esterified) FFAs can, in turn, stimulate FA oxidation; however, notably, they can only yield energy through β-oxidation after esterification and transfer into the mitochondrion, which in turn requires carnitine. High FFA concentrations observed in obesity require high concentrations of carnitine [4]. When acetyl-CoA generation, resulting from β-oxidation or pyruvate oxidation, is greater than its rate of utilisation by the TCA cycle, there is an increase in acetylcarnitine because carnitine buffers excess acetyl-CoA [39]. So, the increase in acetylcarnitine concentration observed in our current study may result from increased production of acetyl-CoA from β-oxidation rather than pyruvate oxidation since serum insulin decreased.

Finally, mannose is a hexose sugar monomer and metabolite of glucose which has a role in protein glycosylation, providing post-translational modifications and structural variance for interaction with other proteins and cells. Mardinoglu et al. reported that plasma mannose concentrations are elevated in individuals with insulin resistance independently of obesity and are strong markers of future risk of T2D and cardiovascular disease [40]. More recently, Ferrannini et al. suggested that mannose could be a marker of insulin resistance, which may be useful for the early identification of individuals with diabetes [41]. However, in our study, we observed a decrease in HOMA-IR and fasting insulin even though serum mannose concentrations were increased post-LED.

Considering other metabolite changes observed in our current study, isobutyrate is a short, volatile, branched-chain fatty acid with a characteristic sweat-like smell. Small amounts of isobutyrate are generated via microbial (gut) metabolism but may also be found in certain foods or fermented beverages [42]. In addition to the endogenous synthesis of SCFAs, Li et al. identified isobutyrate as one of the main volatile compounds in raw milk [43], where the concentration ranges from 1.0 to 15.8 µM [42]. Of relevance to our current findings, isobutyrate has previously been shown to increase following aggressive weight loss achieved through bariatric surgery, with the authors proposing this SCFA to be related to improved insulin sensitivity [33]. Dimethyl sulfone derives from dietary sources, including cow’s milk, from intestinal bacterial metabolism and from human endogenous methanethiol metabolism [44]. According to Xuan and Slupsky, dimethyl sulfone is a common metabolite found in the human metabolome and which is highly influenced by the diet [45]. Plasma choline concentrations respond to dietary intake, where the concentration increases accordingly with the intake of choline and decreases with a choline-inadequate diet [46]. Therefore, it is important to have adequate intake of dietary choline since it is critical for a variety of biological functions and has been shown to be associated with several pathological conditions [46]. Serum choline concentrations in healthy adults were <7.1–20.0 µmol/L [47], and our results of average serum choline concentrations were between 12 µM (week 0) and 9 µM (week 8).

With regard to AA metabolism, a decrease in tyrosine and creatine post-LED was also observed, similar to the studies by Geidenstam et al. and Almanza-Aguilera et al. [14,37], respectively. Newgard et al. showed that weight loss was associated with a decrease in the circulating concentration of seven AAs, including tyrosine [38]. According to Wang et al., tyrosine is strongly associated with an increased risk of developing T2D [48], and according to Gu et al., it acts as a metabolic signal influencing insulin signalling [19]. In addition, Gu et al. found that the decrease in plasma tyrosine concentrations was significantly related to the decrease in HOMA-IR, suggesting that tyrosine may play a crucial role in the development of IR and T2D [19]. Thus, the decrease in tyrosine post-LED, as well as the improvement in HOMA-IR in the current study, may suggest a decrease in the risk of developing T2D. Creatine is positively associated with skeletal muscle mass [49]; therefore, the decrease in creatine post-LED may be directly related to the reduction in FFM that was verified by DXA after the 8 weeks of diet. Likewise, Almanza-Aguilera et al. also found a decrease in creatine after a weight loss intervention based on lifestyle changes [37].

A second goal of this study was to correlate changes in anthropometric and clinical variables with changes in serum metabolite concentrations previously identified. Other than mannose, metabolites associated with lipid metabolism (2-hydroxybutyrate, 3-hydroxybutyrate, *O*-acetylcarnitine and choline) were negatively associated with the change in FM, fasting insulin and TG. Papandreou et al., amongst many others, have previously reported that weight loss and a decrease in adipose mass are associated with changes in lipid metabolism [13]. According to Park et al., lipid profile and FM have been reported as key predictors of metabolic disorders, such as dyslipidemia and T2D [4]. As expected, our current study also showed an improvement in lipid profile, with a decrease in total cholesterol, LDL-cholesterol and TG post-LED and a decrease in FM. Knowing that adipose mass is an important determinant of FFA release into plasma, in which the rate of FFA release from adipose tissue decreases with increasing FM [50], the negative association between a change in FM (%) and a change in 2-hydroxybutyrate, 3-hydroxybutyrate and acetylcarnitine, may be justified. The association of a change in fasting insulin with a change in 3-hydroxybutyrate is in agreement with the results of Geidenstam et al. [14], and this relationship may once again elucidate the increase in FA oxidation and, consequently, in ketogenesis induced by low insulin concentration. Also, the decrease in circulating TG associated with an increase in acetylcarnitine and 2-hydroxybutyrate may be explained by the increase in FA oxidation instead of its esterification [51]. The relationship between the decrease in choline and the increase in circulating TG can be justified by the role that this metabolite plays in lipid metabolism. Phospholipid phosphatidylcholine (derived from choline) is indispensable in the export of TG from the liver to target tissues via a very low-density lipoprotein (VLDL) carrier [46].

Prior analyses from PREVIEW have identified positive behavioural traits in individuals characterised as successful responders to the LED, including more favourable appetite sensations [26]. Hence, finally, it was also an aim of our current study to identify further predictors of LED-induced weight loss, based on the metabolomics analysis. Taking into account the results of other authors [14,16], it was hypothesised that metabolites that predict successful weight loss (≥10%) would be identified. Unexpectedly, however, none of the metabolites assessed were identified as significant predictors of the weight loss. This lack of significant differences could be attributed to several factors. First, the small sample size of our study limits the statistical power to detect subtle changes in metabolite levels. Additionally, our study included only female participants, which may limit the generalisability of the findings to a broader population. Methodological differences between studies can also contribute to these discrepancies. Variations in participant numbers, demographic characteristics and different analytical methodologies used for metabolomics analysis may lead to divergent results. Our analysis focused exclusively on polar metabolites. Including lipid and fatty acid analyses might have provided a more comprehensive view of the metabolic changes and potentially identified significant predictors of weight loss. Therefore, we recommend that future studies include a larger and more diverse participant pool and incorporate a broader range of metabolites, including lipidomics, to better understand the metabolic predictors of weight loss.

## 5. Conclusions

This study demonstrated that an 8-week LED-driven weight loss program in females with overweight and prediabetes improved anthropometric and clinical variables. It also resulted in a change in polar metabolites consistent with a shift in lipid oxidation, with increased serum 2-hydroxybutyrate, 3-hydroxybutyrate and *O*-acetylcarnitine, and decreased choline. Decreased concentrations of creatine, which may be directly related to the observed decrease in FFM, were also observed post-LED, as well as decreased tyrosine concentrations, which may be associated with a decrease in the risk of developing T2D. In addition, increased dimethyl sulfone and isobutyrate were identified post-LED, which may reflect the high intake of low-fat milk used to reconstitute the LED product throughout the 8-week intervention. The correlation between changes in anthropometric and clinical variables with changes in serum metabolite concentrations aligned with the proposed increased lipid oxidation. Unexpectedly, no serum polar metabolites could be identified as potential predictive biomarkers of weight loss success in response to the LED intervention.

## Figures and Tables

**Table 1:** Characteristics of study participants.

	All			Weight Loss (<10%)			Weight Loss (≥10%)		
	Week 0	Week 8	*p*-Value	Week 0	Week 8	*p*-Value	Week 0	Week 8	*p*-Value
Participants, n (%)	28	28		10 (35.7)	10 (35.7)		18 (64.3)	18 (64.3)	
Age (years)	50.5 ± 6.1			50.6 ± 6.8			50.5 ± 5.9		
Height (m)	1.65 ± 0.06			1.65 ± 0.06			1.65 ± 0.07		
Body weight (kg)	100.5 ± 16.3	89.4 ± 14.9	**<0.001**	101.6 ± 13.4	92.6 ± 12.3	**<0.001**	99.9 ± 18.1	87.5 ± 16.3	**<0.001**
BMI (kg/m^2^)	37.0 ± 5.5	31.7 (30.0–35.2) ^†^	**<0.001**	37.3 ± 4.7	34.0 ± 4.3	**<0.001**	36.8 ± 6.1	31.0 (28.3–33.4) ^†^	**<0.001**
Fat-free mass (kg)	51.2 ± 6.1	48.7 ± 5.9	**<0.001**	52.1 ± 5.5	50.3 ± 5.3	**0.014**	50.6 ± 6.5	47.8 ± 6.2	**<0.001**
Fat mass (kg)	48.7 ± 11.1	40.9 ± 10.4	**<0.001**	49.3 ± 9.8	42.6 ± 8.9	**<0.001**	46.4 (39.6–51.5) ^†^	39.9 ± 11.2	**<0.001**
Fat mass (%)	49.6 ± 4.5	46.5 ± 4.9	**<0.001**	49.4 ± 4.5	46.8 ± 4.9	**<0.001**	49.7 ± 4.6	46.3 ± 5.1	**<0.001**
Waist circumference (cm)	108.5 ± 11.7	97.2 ± 10.6	**<0.001**	105.6 ± 7.4	98.3 ± 7.7	**<0.001**	110.2 ± 13.5	96.5 ± 12.0	**<0.001**
Hip circumference (cm)	122.8 ± 10.1	114.2 ± 10.6	**<0.001**	123.9 ± 10.8	115.5 ± 10.2	**<0.001**	122.3 ± 10.0	113.4 ± 11.1	**<0.001**
Waist–hip ratio	0.88 ± 0.06	0.8 (0.8–0.9) ^†^	**0.001**	0.86 ± 0.06	0.85 ± 0.07	0.887	0.90 ± 0.06	0.85 ± 0.04	**<0.001**
FPG (mmol/L)	5.8 ± 0.7	5.8 ± 1.0	0.891	6.2 ± 0.6	6.2 ± 1.5	1.000	5.6 ± 0.6	5.6 ± 0.7	0.820
Fasting insulin (mU/L)	9.8 (7.9–10.7) ^†^	5.3 (4.1–7.9) ^†^	**<0.001**	9.1 ± 2.6	5.3 (4.0–6.8) ^†^	**0.003**	10.3 ± 4.2	6.8 ± 3.6	**<0.001**
HOMA-IR	2.5 ± 0.8	1.3 (1.0–2.2) ^†^	**<0.001**	2.5 ± 0.7	1.7 ± 1.0	**0.006**	2.5 ± 0.9	1.3 (1.0–2.3) ^†^	**0.001**
Systolic BP (mmHg)	122.9 ± 15.7	113.7 ± 11.5	0.006 *	122.7 ± 9.7	115.5 (111.0–118.6) ^†^	0.115	118.2 (109.5–136.4) ^†^	113.6 ± 12.1	0.020 *
Diastolic BP (mmHg)	65.4 ± 8.4	65.1 ± 6.7	0.690 *	68.3 (64.0–72.7) ^†^	66.6 ± 6.0	0.912	64.6 ± 7.8	64.3 ± 7.1	0.727 *
Total cholesterol (mmol/L)	5.4 ± 0.9	4.6 ± 0.8	**<0.001**	5.4 ± 0.7	5.0 ± 0.7	**0.047**	5.4 ± 1.0	4.4 ± 0.8	**<0.001**
HDL-C (mmol/L)	1.4 ± 0.3	1.2 ± 0.3	**<0.001**	1.4 ± 0.3	1.4 ± 0.3	0.540	1.4 ± 0.3	1.1 ± 0.2	**<0.001**
LDL-C (mmol/L)	3.4 ± 0.8	2.9 ± 0.6	**<0.001**	3.4 ± 0.6	3.1 ± 0.6	**0.035**	3.4 ± 0.9	2.8 ± 0.7	**<0.001**
Triglycerides (mmol/L)	1.3 ± 0.5	1.0 ± 0.3	**0.002**	1.3 ± 0.4	1.2 ± 0.4	0.158	1.3 ± 0.5	1.0 ± 0.2	**0.008**

Data expressed as mean ± SD or median (interquartile range) ^†^. Differences between baseline pre- (week 0) and post-LED (week 8) within each group were assessed using paired Student’s *t*-test for variables with normal distribution or the equivalent non-parametric test—Wilcoxon * for variables with non-normal distribution (*p*-value). *p*-values < 0.05 were considered significant (bold). BMI, body mass index; BP, blood pressure; FPG, fasting plasma glucose; HOMA-IR, homeostatic model assessment for insulin resistance; HDL-C, high-density lipoprotein cholesterol; LDL-C, low-density lipoprotein cholesterol.

**Table 2:** Change in anthropometric and clinical outcomes over 8 weeks from pre- (week 0) to post- (week 8) LED in the two weight loss groups.

	All	Weight Loss Groups		Difference between Weight Loss Groups
		<10%	≥10%	*p*-Value
Δ Body weight (kg)	−11.16 ± 2.58	−8.99 ± 1.18	−12.37 ± 2.35	**<0.001**
Δ BMI (kg/m^2^)	−4.12 ± 0.97	−3.30 ± 0.42	−4.57 ± 0.89	**<0.001**
Δ Fat-free mass (kg)	−2.44 ± 1.78	−1.80 ± 1.87	−2.80 ± 1.68	0.16
Δ Fat mass (kg)	−7.84 ± 2.23	−6.75 ± 1.34	−8.44 ± 2.42	0.057
Δ Fat mass (%)	−3.06 ± 1.36	−2.55 ± 1.52	−3.34 ± 1.22	0.382
Δ Waist circumference (cm)	−11.38 ± 5.68	−7.33 ± 4.18	−13.64 ± 5.19	**0.002**
Δ Hip circumference (cm)	−8.63 ± 3.55	−8.33 ± 3.16	−8.81 ± 3.83	0.689
Δ Waist–hip ratio	−0.03 ± 0.04	0.00 ± 0.03	−0.05 ± 0.04	**0.005**
Δ FPG (mmol/L)	−0.03 ± 1.09	0.00 ± 1.52	−0.04 ± 0.82	0.944
Δ Fasting insulin (mU/L)	−3.30 ± 2.79	−3.01 ± 2.33	−3.46 ± 3.07	0.724
Δ HOMA-IR	−0.78 ± 0.75	−0.79 ± 0.70	−0.77 ± 0.79	0.832
Δ Systolic BP (mmHg)	−3.33 (−18.0–1.33) ^†^	−8.83 ± 16.04	−4.67 (−16.83–0.08) ^†^	0.832
Δ Diastolic BP (mmHg)	0.33 (−1.58–3.58) ^†^	−0.37 ± 10.26	0.33 (−1.42–3.33) ^†^	0.724
Δ Total cholesterol (mmol/L)	−0.80 ± 0.69	−0.46 ± 0.63	−0.99 ± 0.65	0.051
Δ HDL-C (mmol/L)	−0.17 ± 0.20	−0.04 ± 0.21	−0.25 ± 0.16	**0.021**
Δ LDL-C (mmol/L)	−0.51 ± 0.51	−0.34 ± 0.43	−0.61 ± 0.53	0.245
Δ Triglycerides (mmol/L)	−0.25 ± 0.40	−0.17 ± 0.35	−0.30 ± 0.42	0.555

Data expressed as mean ± SD or median (interquartile range) ^†^. Changes (Δ) between weight loss groups (<10% vs. ≥10%) were assessed using the non-parametric Mann–Whitney test. *p*-values < 0.05 were considered significant (bold). BMI, body mass index; BP, blood pressure; FPG, fasting plasma glucose; HOMA-IR, homeostatic model assessment for insulin resistance; HDL-C, high-density lipoprotein cholesterol; LDL-C, low-density lipoprotein cholesterol.

**Table 3:** Polar serum metabolite concentrations at pre- (week 0) and post- (week 8) LED time points.

Metabolites (mM)	Week 0	Week 8	*p*-Value
2-Hydroxybutyrate	0.055 ± 0.018	0.070 (0.058–0.092) ^†^	0.002 *
3-Hydroxybutyrate	0.045 (0.033–0.070) ^†^	0.192 (0.106–0.300) ^†^	<0.001 *
Acetate	0.089 ± 0.023	0.095 ± 0.021	0.181
Acetone	0.036 (0.023–0.049) ^†^	0.035 (0.026–0.054) ^†^	0.966
Alanine	0.259 ± 0.078	0.233 ± 0.067	0.082
Betaine	0.032 ± 0.012	0.033 ± 0.012	0.617
Carnitine	0.034 ± 0.010	0.030 ± 0.010	0.060
Choline	0.012 ± 0.004	0.009 ± 0.002	**0.008**
Creatine	0.034 ± 0.016	0.021 ± 0.011	**<0.001**
Creatinine	0.065 ± 0.016	0.070 ± 0.018	0.137
Dimethyl sulfone	0.006 ± 0.003	0.008 ± 0.004	**0.007**
Dimethylamine	0.005 (0.003–0.007) ^†^	0.005 ± 0.002	0.614
Ethanol	0.180 ± 0.017	0.181 ± 0.015	0.661
Formate	0.416 ± 0.085	0.417 ± 0.076	0.956
Glycerol	0.112 ± 0.045	0.114 ± 0.048	0.897
Histidine	0.046 ± 0.013	0.042 (0.040–0.057) ^†^	0.994
Isobutyrate	0.008 ± 0.002	0.010 ± 0.003	0.006 *
Isoleucine	0.048 ± 0.014	0.051 ± 0.016	0.609
Lactate	2.297 ± 0.965	1.982 ± 0.659	0.066
Leucine	0.102 ± 0.028	0.102 ± 0.027	0.990
Lysine	0.055 ± 0.017	0.052 ± 0.013	0.357
Mannose	0.047 ± 0.013	0.056 ± 0.015	**0.003**
Methanol	0.275 ± 0.020	0.268 (0.259–0.276) ^†^	0.690
Methionine	0.021 ± 0.006	0.021 ± 0.006	0.695
Methylamine	0.018 ± 0.005	0.021 ± 0.006	0.111
*O*-Acetylcarnitine	0.007 ± 0.002	0.010 (0.009–0.014) ^†^	<0.001 *
*O*-Phosphocholine	0.003 (0.001–0.004) ^†^	0.003 ± 0.002	0.289
Phenylalanine	0.045 ± 0.014	0.041 ± 0.010	0.174
Pyruvate	0.047 ± 0.019	0.039 ± 0.018	0.053
Threonine	0.111 ± 0.043	0.106 ± 0.030	0.646
Trimethylamine *N*-oxide	0.005 (0.003–0.007) ^†^	0.005 ± 0.002	0.531 *
Tryptophan	0.046 ± 0.012	0.045 ± 0.011	0.868
Tyrosine	0.058 ± 0.016	0.048 ± 0.013	**0.005**
Valine	0.184 ± 0.053	0.180 ± 0.040	0.716

Data expressed as mean ± SD or median (interquartile range) ^†^. Differences between pre- (week 0) and post-LED (week 8) were assessed using paired Student’s *t*-test (for variables with normal distribution) or the equivalent non-parametric test—Wilcoxon * (for variables with non-normal distribution). *p*-values < 0.05 were considered significant (bold).

**Table 4:** Association between change in clinical variables and change in serum metabolite concentrations.

	**Δ 2-Hydroxybutyrate**	***p*-Value**	**Δ 3-Hydroxybutyrate**	***p*-Value**	**Δ Choline**	***p*-Value**	**Δ Creatine**	***p*-Value**	**Δ Dimethyl Sulfone**	***p*-Value**
Δ BMI (kg/m^2^)	−0.05 (−16.62; 16.52)	0.995	−35.99 (−101.53; 29.54)	0.282	0.26 (−2.16; 2.68)	0.833	0.63 (−6.02; 7.28)	0.853	0.79 (−0.62; 2.20)	0.271
Δ FM (%)	**−6.10** **(−11.62; −0.59)**	**0.030**	**−36.66** **(−70.28; −3.05)**	**0.033**	−1.60 (−3.79; 0.60)	0.154	−1.97 (−6.61; 2.68)	0.406	−0.52 (−1.62; 0.58)	0.355
Δ Fasting insulin (mU/L)	−3.00 (−6.08; 0.07)	0.055	**−23.19** **(−35.53; −10.85)**	**< 0.001**	−0.35 (−1.18; 0.47)	0.399	0.08 (−1.91; 2.06)	0.941	−0.13 (−0.56; 0.30)	0.550
Δ HDL-C (mmol/L)	−1.97 (−81.26; 77.32)	0.961	−90.85 (−500.61; 318.90)	0.664	1.67 (−16.10; 19.45)	0.854	15.38 (−23.23; 53.99)	0.435	−1.35 (−12.64; 9.94)	0.815
Δ TG (mmol/L)	**−34.27** **(−65.98; −2.56)**	**0.034**	−160.16 (−366.38; 46.06)	0.128	**−4.80** **(−8.61; −0.99)**	**0.014**	−7.06 (−24.63; 10.51)	0.431	−2.13 (−5.35; 1.08)	0.193
Δ Waist circumference (cm)	−0.39 (−2.73; 1.95)	0.746	−4.25 (−21.26; 12.77)	0.625	0.09 (−0.37; 0.56)	0.692	0.54 (−0.93; 2.01)	0.472	0.15 (−0.09; 0.39)	0.223
	**Δ** **Isobutyrate**	***p*-Value**	**Δ** **Mannose**	***p*-Value**	**Δ** ***O*-Acetylcarnitine**		***p*-Value**		**Δ** **Tyrosine**	***p*-Value**
Δ BMI (kg/m^2^)	0.06 (−1.12; 1.23)	0.924	−5.41 (−13.19; 2.38)	0.173	−0.46 (−2.44; 1.53)		0.652		3.23 (−2.71; 9.16)	0.287
Δ FM (%)	−0.69 (−1.66; 0.27)	0.158	**−4.13** **(−8.06; −0.19)**	**0.040**	**−1.23** **(−2.16; −0.30)**		**0.010**		−3.40 (−7.69; 0.89)	0.120
Δ Fasting insulin (mU/L)	0.18 (−0.17; 0.53)	0.316	0.60 (−1.42; 2.63)	0.558	−0.23 (−0.73; 0.26)		0.358		0.74 (−1.42; 2.89)	0.504
Δ HDL-C (mmol/L)	−0.95 (−8.33; 6.43)	0.801	−2.76 (−25.97; 20.44)	0.815	2.45 (−10.05; 14.95)		0.700		14.06 (−30.29; 58.42)	0.534
Δ TG (mmol/L)	−1.31 (−5.31; 2.69)	0.521	−13.46 (−34.78; 7.86)	0.216	**−7.19** **(−13.08; −1.30)**		**0.017**		−14.96 (−58.72; 28.81)	0.503
Δ Waist circumference (cm)	−0.04 (−0.27; 0.19)	0.735	0.10 (−0.84; 1.04)	0.832	0.03 (−0.39; 0.44)		0.899		0.38 (−1.83; 2.59)	0.736

Data expressed as beta estimate (β) and 95% confidence intervals, adjusted for age, in µM. Change (Δ) represents variation in clinical independent variables between pre- (week 0) and post-LED (week 8). Significant values are presented in bold. BMI, body mass index; FM, fat mass; HDL-C, high-density lipoprotein cholesterol; TG, triglycerides.

**Table 5:** Metabolite concentrations (µM) at baseline week 0 associated with ≥10% weight loss.

	Model 1		Model 2	
	β (95% CI)	*p*-Value	β (95% CI)	*p*-Value
2-Hydroxybutyrate	1.5 (−41.19; 44.09)	0.947	2.3 (−47.18; 51.80)	0.927
3-Hydroxybutyrate	−8.6 (−29.24; 12.04)	0.414	−10.8 (−43.33; 21.66)	0.513
Acetate	25.5 (−12.75; 63.76)	0.191	27.0 (−15.63; 69.65)	0.214
Acetone	16.3 (−21.68; 54.43)	0.399	23.5 (−25.25; 72.21)	0.345
Alanine	3.4 (−6.93; 13.67)	0.521	5.3 (−6.30; 16.91)	0.370
Betaine	24.8 (−44.22; 93.72)	0.482	40.5 (−54.28; 135.24)	0.402
Carnitine	−2.3 (−78.06; 73.42)	0.952	27.1 (−64.75; 118.86)	0.564
Choline	−16.8 (−207.99; 174.33)	0.863	15.4 (−207.20; 238.04)	0.892
Creatine	10.3 (−40.45; 60.97)	0.692	0.9 (−62.08; 63.93)	0.977
Creatinine	14.8 (−34.75; 64.41)	0.558	30.2 (−28.80; 89.25)	0.316
Dimethyl sulfone	62.9 (−205.97; 331.80)	0.647	91.1 (−260.56; 442.73)	0.612
Dimethylamine	157.6 (−157.10; 472.30)	0.326	244.4 (−175.07; 663.80)	0.254
Ethanol	7.1 (−40.01; 54.10)	0.769	−17.9 (−74.55; 38.72)	0.535
Formate	3.5 (−6.02; 13.07)	0.470	5.5 (−5.53; 16.49)	0.329
Glycerol	−15.7 (−35.13; 3.80)	0.115	−14.3 (−37.63; 8.97)	0.228
Histidine	30.3 (−31.61; 92.11)	0.338	32.1 (−38.38; 102.48)	0.372
Isobutyrate	61.0 (−255.69; 377.70)	0.706	58.0 (−307.51; 423.47)	0.756
Isoleucine	29.7 (−29.49; 88.94)	0.325	33.8 (−30.92; 98.53)	0.306
Lactate	0.4 (−0.53; 1.23)	0.432	0.4 (−0.54; 1.36)	0.393
Leucine	14.7 (−13.51; 42.82)	0.308	14.9 (−16.44; 46.15)	0.352
Lysine	15.1 (−32.74; 62.89)	0.537	10.9 (−40.25; 61.98)	0.677
Mannose	−17.8 (−81.19; 45.51)	0.581	−24.0 (−92.75; 44.78)	0.494
Methanol	12.4 (−29.84; 54.66)	0.565	17.9 (−33.45; 69.31)	0.494
Methionine	72.4 (−65.33; 210.03)	0.303	100.0 (−68.22; 267.26)	0.245
Methylamine	100.2 (−65.18; 265.50)	0.235	65.7 (−111.27; 242.66)	0.467
*O*-Acetylcarnitine	−82.1 (−402.74; 238.49)	0.616	−68.4 (−460.72; 323.84)	0.732
*O*-Phosphocholine	−44.4 (−372.31; 283.45)	0.791	−123.1 (−502.88; 256.63)	0.525
Phenylalanine	16.8 (−41.12; 74.74)	0.570	21.0 (−44.84; 86.74)	0.533
Pyruvate	23.7 (−22.06; 69.46)	0.310	14.9 (−32.15; 62.00)	0.534
Threonine	6.5 (−12.40; 25.30)	0.502	8.6 (−17.45; 34.61)	0.518
Trimethylamine *N*-oxide	30.0 (−112.81; 172.77)	0.681	0.7 (−196.31; 197.61)	0.995
Tryptophan	9.0 (−54.52; 72.51)	0.781	19.1 (−52.69; 90.79)	0.603
Tyrosine	27.8 (−22.42; 77.92)	0.278	26.6 (−28.70; 81.98)	0.345
Valine	6.1 (−8.79; 21.02)	0.422	6.8 (−9.18; 22.81)	0.403

Model 1: non-adjusted coefficient; Model 2: coefficient adjusted for body weight, fasting insulin, HDL-cholesterol, TG and waist circumference at baseline, week 0.

## Data Availability

The original contributions presented in the study are included in the article/Supplementary Material, further inquiries can be directed to the corresponding author/s.

## Supplementary Materials

The following supporting information can be downloaded at: https://www.mdpi.com/article/10.3390/metabo14080401/s1, Table S1—Observed ^1^H and ^13^C NMR chemical shifts for quantified metabolites in pooled serum samples; Figure S1—Score Plot of PLS-DA of all paired samples and results of cross-validation.
