# Supplementary material for: Changes in Serum Metabolome Following Low-Energy Diet-Induced Weight Loss in Women with Overweight and Prediabetes: A PREVIEW-New Zealand Sub-Study
Source: Metabolites. 2024 Jul 24;14(8):401. doi: 10.3390/metabo14080401 (PMC11356139; doi:10.3390/metabo14080401)
Supplement: Supplementary file 1 [file metabolites-14-00401-s001.zip › metabolites-3081686-supplementary.pdf]

Supplementary materials

for

Changes in Serum Metabolome Following Low-Energy Diet-Induced Weight Loss in Women with Overweight and Prediabetes: A PREVIEW-New Zealand Sub-Study

Bárbara Relva [1], Linda M. Samuelsson [2], Iola F. Duarte [3], Ulrike Fasol [4], Patrick J. B. Edwards [5], Mikael Fogelholm [6], Anne Raben [7,8], Sally D. Poppitt [9,*,†] and Marta P. Silvestre [1,9,10,*,†]

1 NOVA Medical School, Faculdade de Ciências Médicas, NMS|FCM, Universidade Nova de Lisboa, 1169-056 Lisboa, Portugal; barbara.c.relva@edu.nms.unl.pt
2 Smart Foods & Bioproducts Group, AgResearch Ltd., Palmerston North 4442, New Zealand; linda.samuelsson@agresearch.co.nz
3 Department of Chemistry, CICECO-Aveiro Institute of Materials, University of Aveiro, 3810-193 Aveiro, Portugal; ioladuarte@ua.pt
4 Faculty of Medical and Life Sciences, Furtwangen University, 78054 Villingen-Schwenningen, Germany; ulrike.fasol@hs-furtwangen.de
5 School of Natural Sciences, Massey University, Palmerston North 4442, New Zealand; p.j.edwards@massey.ac.nz
6 Department of Food and Nutrition, University of Helsinki, Unioninkatu 44, 00014 Helsinki, Finland; mikael.fogelholm@helsinki.fi
7 Department of Nutrition, Exercise and Sports, University of Copenhagen, Nørre Allé 51, DK-2200 Copenhagen, Denmark; ara@nexs.ku.dk
8 Department for Clinical and Translational Research, Copenhagen University Hospital, Steno Diabetes Center Copenhagen, Blegdamsvej 9, DK-2100 Herlev, Denmark
9 Human Nutrition Unit, School of Biological Sciences, Department of Medicine, University of Auckland, 18 Carrick Place, Auckland 1024, New Zealand
10 CINTESIS@RISE, NOVA Medical School, Faculdade de Ciências Médicas, NMS|FCM, Universidade Nova de Lisboa, 1169-056 Lisboa, Portugal
* Correspondence: s.poppitt@auckland.ac.nz (S.D.P.); marta.silvestre@nms.unl.pt (M.P.S.)
† The authors contributed equally to this work.

Table S1 - Observed ^{1}H and ^{13}C NMR chemical shifts (d) for quantified metabolites (referenced to DSS at 0.00 ppm) in pooled serum samples.

Metabolite	^{1}H chemical shift (ppm)	^{13}C chemical shift (ppm)
**2-Hydroxybutyrate**	0.88	11.3
	1.66	29.8
	1.70	29.3
	3.99	76.2
**3-Hydroxybutyrate**	1.19	24.5
	2.30	49.2
	2.39	49.3
	4.14	68.5
**Acetate**	1.91	25.9
**Acetone**	2.22	32.9
**Alanine**	1.46	19.1

	3.76	53.4
**Betaine**	3.25	54.9
	3.89	68.8
**Carnitine**	2.42	45.6
	3.22	56.7
	3.44	73.1
	4.55	66.4
**Choline**	3.19[1]	56.6[1]
	3.51	70.3
	4.07	58.8
**Creatine**	3.02	39.7
	3.92	56.6
**Creatinine**	3.03	33.0
	4.05	59.1
**Dimethyl sulfone**	3.14	-[2]
**Dimethylamine**	2.70	36.9
**Ethanol**	1.17	19.6
	3.64	60.3
**Formate**	8.44	
**Glycerol**	3.56	65.3
	3.64	65.3
	3.78	74.9
**Histidine**	3.04	31.5
	3.18	31.6
	3.90	57.8
	7.01	120.8
	7.72	139.2
**Isobutyrate**	1.07	21.8
	2.38	39.9
**Isoleucine**	0.92	13.8
	1.00	17.3
	1.24	27.2
	1.46	27.2
	1.95	38.8
	3.64	62.5
**Lactate**	1.31	22.8
	4.10	71.8
**Leucine**	0.94	23.7
	0.95	24.8
	1.66	42.8
	1.67	37.6
	1.70	42.8
	3.70	56.4
**Lysine**	1.43	23.9
	1.48	23.9
	1.70	29.3
	1.86	33.2
	3.02	42.1
	3.68	57.3
**Mannose (α + β)[3]**	3.36	78.8
	3.57	69.7

	3.65	69.7
	3.76	59.5
	3.84	73.1
	3.88	59.3
	3.91	59.0
	3.92	73.6
	5.17	96.8
**Methanol**	3.35	51.7
**Methionine**	2.10	18.0
	2.16	32.4
	2.62	31.6
	3.78	56.9
**Methylamine**	2.60	27.0
***O*-Acetylcarnitine**	2.13	23.3
	2.51	43.1
	2.60	43.0
	3.18[1]	56.5[1]
	3.58	71.0
	3.85	71.4
	5.59	69.9
***O*-Phosphocholine**	3.21	56.0
	3.58	68.7
	4.16	61.0
**Phenylalanine**	3.08	38.9
	3.23	39.0
	3.91	59.1
	7.31	132.1
	7.35	130.3
	7.41	131.8
**Pyruvate**	2.36	29.3
**Threonine**	1.30	22.1
	3.54	64.4
	4.19	69.4
**Trimethylamine *N*-oxide**	3.25	62.3
**Tryptophan**	3.28	29.1
	3.45	29.3
	4.00	58.0
	7.20	122.2
	7.27	124.8
	7.30	128.0
	7.53	114.8
	7.72	121.2
**Tyrosine**	3.00	38.4
	3.15	38.9
	3.88	59.3
	6.86	118.7
	7.17	133.5
**Valine**	0.97	19.3
	1.02	20.7
	2.25	32.0
	3.57	63.4

[1]Cross peaks from choline and *O*-acetylcarnitine overlap.

[2]No cross peak was detected in the HSQC.

[3]Not all cross peaks for the α and β forms of mannose were detected. For instance, the cross peak from the anomeric proton in β-mannose was obscured by the large water peak.

(a)

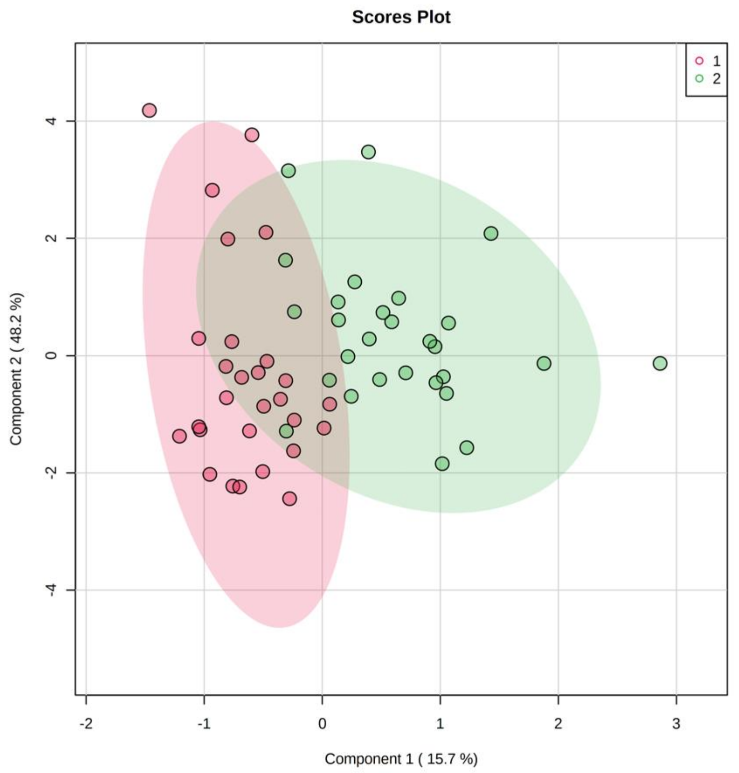

(**b**)

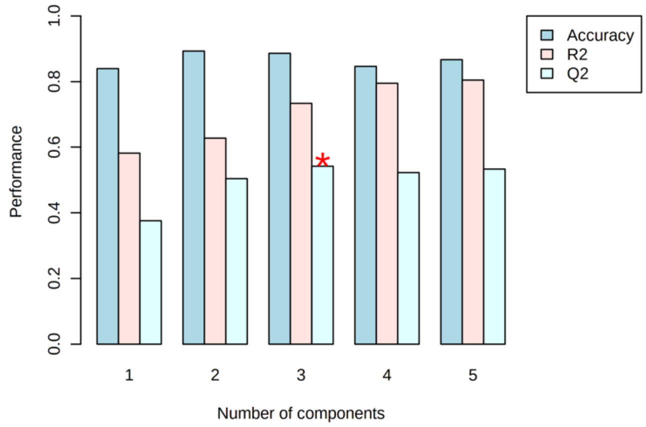

Figure S1 - (**a**) Scores Plot of PLS-DA of all paired samples and (**b**) results of cross validation. Red symbols - CD1, Green symbols - CD2
